# Bioenergetic bypass using cell-permeable succinate, but not methylene blue, attenuates metformin-induced lactate production

**DOI:** 10.1186/s40635-018-0186-1

**Published:** 2018-08-01

**Authors:** Sarah Piel, Johannes K. Ehinger, Imen Chamkha, Eleonor Åsander Frostner, Fredrik Sjövall, Eskil Elmér, Magnus J. Hansson

**Affiliations:** 1 Department of Clinical Sciences Lund, Mitochondrial Medicine, Lund University, BMC A13, 22184 Lund, Sweden; 2 NeuroVive Pharmaceutical AB, Medicon Village, 22381 Lund, Sweden; 3 Department of Clinical Sciences Lund, Otorhinolaryngology, Head and Neck Surgery, Lund University, Skane University Hospital, 22185 Lund, Sweden; 4 Department of Clinical Sciences Lund, Intensive Care and Perioperative Medicine, Lund University, Skane University Hospital, 20502 Malmö, Sweden; 5 Department of Clinical Sciences Lund, Clinical Neurophysiology, Lund University, Skane University Hospital, 22185 Lund, Sweden

**Keywords:** Cell-permeable succinate, Human platelets, Lactic acidosis, Metformin, Methylene blue, Mitochondrial respiration, Mitochondrial toxicity

## Abstract

**Background:**

Metformin is the most common pharmacological treatment for type 2 diabetes. It is considered safe but has been associated with the development of lactic acidosis under circumstances where plasma concentrations exceed therapeutic levels. Metformin-induced lactic acidosis has been linked to the drug’s toxic effect on mitochondrial function. Current treatment strategies aim to remove the drug and correct for the acidosis. With a mortality of 20%, complementary treatment strategies are needed. In this study, it was investigated whether targeting mitochondria with pharmacological agents that bypass metformin-induced mitochondrial dysfunction can counteract the energetic deficit linked to toxic doses of metformin.

**Methods:**

The redox agent methylene blue and the cell-permeable succinate prodrug NV118 were evaluated by measuring mitochondrial respiration and lactate production of human platelets exposed to metformin and co-treated with either of the two pharmacological bypass agents.

**Results:**

The cell-permeable succinate prodrug NV118 increased mitochondrial respiration which was linked to phosphorylation by the ATP-synthase and alleviated the increase in lactate production induced by toxic doses of metformin. The redox agent methylene blue, in contrast, failed to mitigate the metformin-induced changes in mitochondrial respiration and lactate generation.

**Conclusions:**

The cell-permeable succinate prodrug NV118 bypassed the mitochondrial dysfunction and counteracted the energy deficit associated with toxic doses of metformin. If similar effects of NV118 prove translatable to an in vivo effect, this pharmacological strategy presents as a promising complementary treatment for patients with metformin-induced lactic acidosis.

**Electronic supplementary material:**

The online version of this article (10.1186/s40635-018-0186-1) contains supplementary material, which is available to authorized users.

## Background

Metformin is the most common pharmacological treatment for type 2 diabetes [1]. Downregulation of hepatic gluconeogenesis and decreased glucose uptake through the gut, partially due to drug-induced decreased mitochondrial function, are key aspects of metformin’s antidiabetic effect. However, the exact mechanisms are not fully elucidated yet [2]. Metformin is considered safe but has been associated with cases of lactic acidosis. Lactic acidosis is defined as arterial lactate levels above 5 mM and a pH below 7.35 [1, 2]. Metformin-induced lactic acidosis (MILA) appears primarily in patients with renal failure, circumstances under which the drug’s blood concentration can exceed therapeutic levels. Pathological conditions affecting cardiovascular, respiratory, and liver function can further exacerbate the lactic acidosis as they can impair lactate metabolism and/or the acid-base balance [1, 3, 4]. Not surprisingly, the inhibitory action of metformin on mitochondrial function is also implicated in the development of MILA. Metformin inhibits the mitochondrial glycerophosphate dehydrogenase (mGPD) and complex I (CI) of the oxidative phosphorylation (OXPHOS) pathway [2, 5]. As a result, the cell increases glycolysis to compensate for the loss of mitochondrial ATP production, which is associated with increased lactate production and acidification. Current treatment strategies consist of supportive measures, forced clearance to remove the drug and correction of the acidosis [3]. With a mortality of around 20% [6, 7], there is a need for complementary treatment strategies for patients with MILA.

In this study, we hypothesized that restoration of the OXPHOS pathway by targeting mitochondria with pharmacological agents that bypass metformin-induced mitochondrial dysfunction can alleviate the changes in mitochondrial energy production associated with metformin intoxication. We investigated the redox agent methylene blue (MB), which has been described to facilitate electron transfer from NAD(P)H-dependent dehydrogenases to cytochrome C [8–12], and the cell-permeable succinate prodrug NV118, which readily passes through the cell membrane independent of active transporters, releases succinate, and, through oxidation at complex II (CII), donates electrons to the OXPHOS pathway [13]. Our hypothesis was tested by measuring mitochondrial respiration and lactate production of human platelets exposed to metformin and co-treated with either of the two pharmacological bypass agents.

## Methods

### Materials

All chemicals were obtained from Sigma-Aldrich Chemie GmbH (Schnelldorf, Germany). The cell-permeable succinate prodrugs were supplied by NeuroVive Pharmaceutical AB (Lund, Sweden) [13], and MB was purchased from Sigma-Aldrich Chemie GmbH (Schnelldorf, Germany).

### Human platelet isolation

Venous blood from healthy volunteers was drawn in K_2_EDTA tubes (Vacutainer®, BD, Franklin Lakes, USA), and human platelets were isolated as previously described [14].

### High-resolution respirometry

High-resolution respirometry of human platelets was performed as previously described [5]. The corresponding doses of MB and NV118 used for further evaluation were determined based on dose-response experiments performed in rotenone-intoxicated human platelets. Both bypass strategies were then evaluated in a model of (a) CI inhibition caused by rotenone (2 μM) and (b) specific metformin-related mitochondrial dysfunction induced by exposure to either 10 mM metformin or 50 mM metformin for 60 min [5]. In the model of rotenone intoxication, MB was evaluated at 20 μM. In the model of metformin-induced mitochondrial dysfunction, the dose of MB was adjusted to 10 μM based on an additional dose-response performed with the lactate production assay. NV118 was evaluated in both models at 250 μM. Mitochondrial respiration coupled to phosphorylation, here referred to as *coupled respiration*, was evaluated by addition of the ATP-synthase inhibitor oligomycin (1 μg/ml) to block the phosphorylation pathway, and calculated as the difference in respiration before and after the inhibition of the ATP-synthase. Control experiments were performed without the addition of oligomycin to account for background drift of oxygen consumption. Complex III (CIII) was blocked with antimycin A (1 μg/ml), and complex IV (CIV) was inhibited with sodium azide (10 mM). Remaining respiration after addition of sodium azide was defined as non-mitochondrial respiration.

**Fig. 1:**
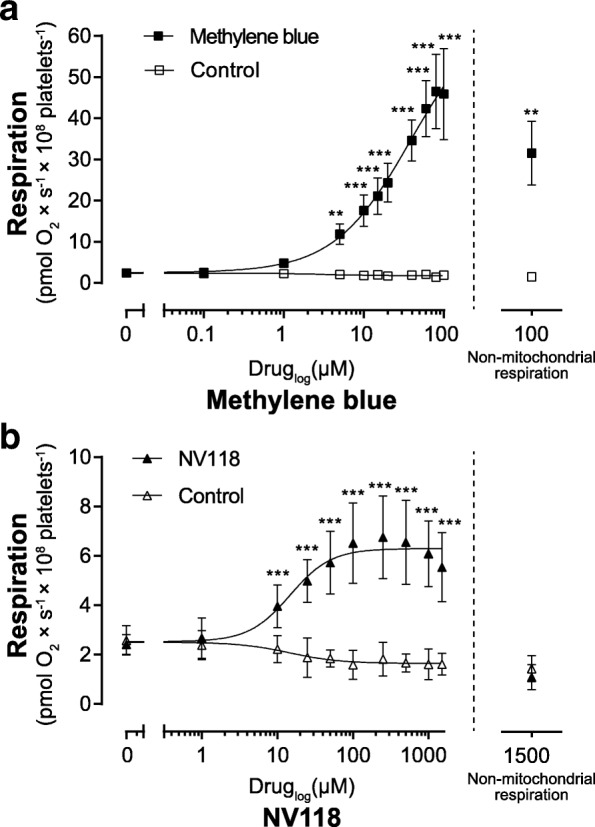
Dose-response of methylene blue and NV118 on oxygen consumption in rotenone-intoxicated human platelets. Respiration was measured in human platelets with complex I inhibition induced by rotenone (2 μM). The potential of the pharmacological bypass strategies methylene blue (**a** black square) and the cell-permeable succinate prodrug NV118 (**b** black triangle) to increase rotenone-inhibited respiration was evaluated by titrating increasing doses of drug or vehicle (**a** white square, double-deionized water; **b** white triangle, DMSO). After maximal respiration was reached, the contribution of non-mitochondrial respiration to total respiration was evaluated by addition of the complex III inhibitor antimycin A (1 μg/ml) followed by the complex IV inhibitor sodium azide (10 mM). The residual respiration shows the non-mitochondrial respiration at the highest dose of each drug. Data are expressed as mean ± SD. Non-linear curve fitting was applied for generation of the dose-response curves. *n* = 4. Two-way ANOVA with Bonferroni post hoc test was performed for analysis of differences. **p < 0.01, ***p < 0.001, compared to vehicle control

**Fig. 2:**
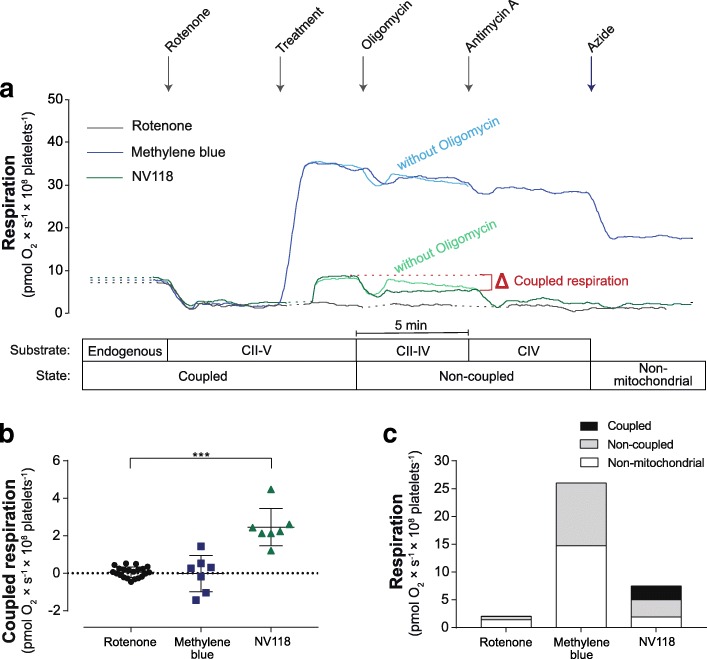
Effect of methylene blue and NV118 on coupled respiration in rotenone-intoxicated human platelets. **a** Representative traces of mitochondrial respiration in human platelets with complex I inhibition induced by rotenone (2 μM). Mitochondrial respiration due to coupled phosphorylation of rotenone-intoxicated platelets treated with vehicle (rotenone, dark gray trace), methylene blue (20 μM, dark blue trace) or the cell-permeable succinate prodrug NV118 (250 μM, dark green trace), here referred to as coupled respiration, was evaluated by subsequent addition of the ATP-synthase inhibitor oligomycin (1 μg/ml) to block the phosphorylation pathway, and calculated as the difference in respiration before and after the inhibition of the ATP-synthase. The protocol was continued by adding the complex III inhibitor antimycin A (1 μg/ml) followed by the complex IV inhibitor sodium azide (10 mM). Control experiments were performed without the addition of oligomycin to account for background drift of oxygen consumption in the presence of the bypass agents (light blue trace, light green trace). **b** Quantification of coupled respiration of rotenone-intoxicated platelets treated with vehicle (rotenone, black circle), methylene blue (20 μM, dark blue square), or the cell-permeable succinate prodrug NV118 (250 μM, dark green triangle) was calculated as the difference in respiration before and after inhibition of the ATP-synthase. **c** The coupled respiration, non-coupled respiration, and non-mitochondrial respiration was evaluated in rotenone-intoxicated platelets treated with vehicle, methylene blue, or NV118. Non-mitochondrial respiration was defined as respiration remaining after addition of sodium azide, which all other respiratory values were corrected for. Data are expressed as individual scatter plot and mean ± SD (**b**) or mean (**c**). Rotenone: *n* = 21, NV118 and methylene blue: *n* = 7. One-way ANOVA with Dunnet post hoc test was used for comparison of the drug’s effect on coupled respiration. ***p < 0.001. CII–V: complex II to V, CII–IV: complex II to IV, CIV: complex IV

To exclude effects on mitochondrial respiration induced by the vehicles of the bypass strategies (NV118, DMSO; MB, double-deionized water), the effects of the corresponding vehicles were evaluated simultaneously (Fig. 1) or in a separately performed experiment (Fig. 2, vehicle controls of both bypass agents were pooled). In the model of specific metformin-related mitochondrial dysfunction, a vehicle control (double-deionized water) to the metformin treatment was included.

### Lactate production

Platelets were incubated with metformin (10 mM) or its vehicle (double-deionized water) in glucose-containing PBS (10 mM) for 60 min before co-treatment with either MB or the cell-permeable succinate prodrug was initiated. MB was administered either as a single dose (10 μM) or as one dose every 30 min (1 μM). The cell-permeable succinate prodrugs NV118, NV189, and NV241, or succinate, were given every 30 min (250 μM). Lactate levels in the medium were measured every 30 min over 4 h, and the lactate production was calculated from onset of intervention (60–240 min) as previously described [13]. Vehicle control and metformin alone were run on each occasion.

### Data analysis

Dose-response evaluation of MB and NV118 on respiration in rotenone-intoxicated human platelets was performed with a group size of four replicates (*n* = 4). As the magnitude of the change in the evaluated parameter was not pre-specified, power calculation for sample size was not applied here. Unless indicated otherwise, all other evaluations were performed with a sample size of seven replicates (*n* = *7*). This sample size was calculated from previously published data showing increased lactate production by human platelets exposed to metformin (10 mM) [5] and determined to be required to detect a targeted 25% reduction in lactate production in human platelets exposed to metformin (10 mM) for 4 h and co-treated with either pharmacological treatment, an alpha level of 5% and power of 80%. Data are expressed as scatter plot or mean ± SD. Lactate production was calculated using standard non-linear curve fitting. Statistical analysis was performed using GraphPad Prism version 7 (GraphPad Software, Inc., La Jolla, California, USA). Data from blood cell respirometry have been reported to be normally distributed [14], and parametric tests were used for analyses of differences. For multiple comparisons of two groups (Fig. 1), two-way ANOVA with Bonferroni post hoc test was performed. One-way ANOVA with Dunnet post hoc test was applied for one-factor comparison of three or more groups. A *p* value of 0.05 or less was considered to indicate significant difference. No blinding or randomization was performed.

## Results

### Dose-response of methylene blue and NV118 on respiration in rotenone-intoxicated human platelets

MB and NV118 dose dependently increased respiration in human platelets with rotenone-induced CI inhibition (Fig. 1). MB started to increase respiration at 5 μM (*p* < 0.01) and reached the maximum with a 33-fold increase at 80 μM (*p* < 0.001) as compared to control. MB also induced non-mitochondrial respiration which, at the highest concentration investigated here, was higher than control (*p* < 0.01), and responsible for 69% of total respiration (Fig. 1a). NV118 started to increase respiration at 10 μM (*p* < 0.001) with a maximum and fourfold increase at 250 μM (*p* < 0.001) and displayed no effect on non-mitochondrial respiration (Fig. 1b). Based on the dose-response and effect on non-mitochondrial respiration, 20 μM MB (15-fold increase compared to control) and 250 μM NV118 were selected for further evaluation in the model of rotenone intoxication. Neither of the vehicles of the pharmacological bypass strategies increased mitochondrial respiration (Fig. 1).

### Effect of methylene blue and NV118 on coupled respiration in rotenone-intoxicated human platelets

Both MB and NV118 increased respiration in rotenone-intoxicated human platelets as compared to vehicle-treated controls (Fig. 2a). The increase in respiration with MB was not caused by increased coupled respiration (rotenone = 0.05 pmol O_2_ × s^−1^ × 10^8^ platelets^−1^; MB = − 0.02 pmol O_2_ × s^−1^ × 10^8^ platelets^−1^; *p* = 0.95) (Fig. 2b) but elevated non-coupled and non-mitochondrial respiration, accounting for 43.5 and 56.5% of total, drug-induced respiration respectively (Fig. 2a, c). NV118, in contrast, increased coupled respiration significantly as compare to vehicle-treated, rotenone-intoxicated human platelets (NV118 = 2.46 pmol O_2_ × s^−1^ × 10^8^ platelets^−1^; *p* < 0.001) (Fig. 2b), accounting for 32.9% of total, drug-induced respiration and with no effect on non-mitochondrial respiration (Fig. 2a, c).

### Effect of methylene blue and NV118 on coupled respiration and lactate metabolism in metformin-induced mitochondrial dysfunction in human platelets

**Fig. 3:**
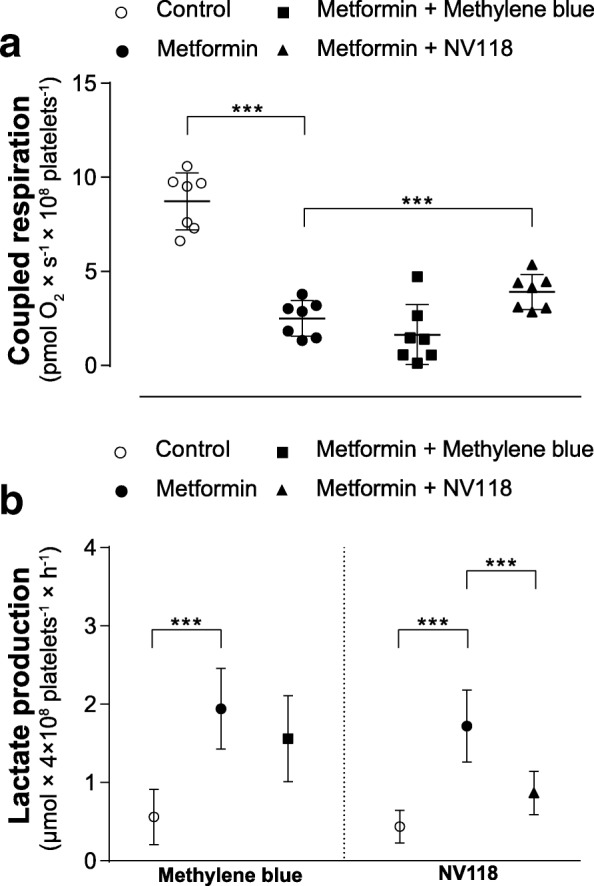
Effect of methylene blue and NV118 on coupled respiration and lactate production in metformin-intoxicated human platelets. **a** Mitochondrial respiration was measured in human platelets with mitochondrial dysfunction induced by 60 min exposure to metformin (black circle; 50 mM). After subsequent addition of methylene blue (10 μM, black square) or the cell-permeable succinate prodrug NV118 (250 μM, black triangle), mitochondrial respiration due to coupled phosphorylation, here referred to as coupled respiration, was evaluated by addition of the ATP-synthase inhibitor oligomycin (1 μg/ml) to block the phosphorylation pathway, and calculated as the difference in respiration before and after the inhibition of the ATP-synthase. Subsequently, the complex III inhibitor antimycin A (1 μg/ml) followed by the complex IV inhibitor sodium azide (10 mM) were added. Control experiments were performed without the addition of oligomycin to account for background drift of oxygen consumption. A vehicle control to metformin was run with each experiment (white circle). **b** Lactate production of human platelets incubated with metformin (10 mM, black circle) was measured every 30 min over 4 h with or without co-treatment of methylene blue (10 μM, single dose, black square) or NV118 (250 μM, every 30 min, black triangle) starting at 60 min. A vehicle control was run with each experiment (white circle). Data are expressed as individual scatter plot and mean ± SD (**a**) or mean ± SD (**b**). All experiments were performed with *n* = 7, with exception of the control and metformin group of the lactate production assay run with methylene blue (*n* = 9) (**b**). One-way ANOVA with Dunnet post hoc test was used. ***p < 0.001, compared to metformin

Both 10 mM metformin (− 28%, *p* < 0.05) (Additional file 1) and 50 mM metformin (− 69%, *p* < 0.001) (Fig. 3a) reduced coupled respiration in human platelets significantly compared to control. Samples treated with MB showed a tendency towards decreased coupled respiration compared to metformin alone (10 mM metformin: − 13%, *p* = 0.49; 50 mM metformin: − 32%, *p* = 0.41) (Additional file 1, Fig. 3a). NV118, to the contrary, increased coupled respiration by 20% after exposure to 10 mM metformin (*p* = 0.37) (Additional file 1) and by 46% after exposure to 50 mM metformin (*p* < 0.001) (Fig. 3a) as compared to metformin alone.

Metformin significantly increased lactate production compared to vehicle control (*p* < 0.001) (Fig. 3b). Co-treatment with a single dose of MB (10 μM) did not reduce lactate production significantly (− 20%, *p* = 0.30) (Fig. 3b) and neither did supplementation with a low dose of MB (1 μM) every 30 min (− 11%, *p* = 0.69) (Additional file 2). In contrast, co-treatment with NV118 (250 μM) every 30 min alleviated the metformin-induced increase in lactate production significantly (− 50%, *p* < 0.001) (Fig. 3b). Qualitatively, similar results were obtained for NV189 and NV241, two compounds of the same drug class as NV118, whereas succinate did not mitigate the metformin-induced increase in lactate production (Additional file 2).

### Bypass mechanism of methylene blue and NV118 in human platelets with rotenone intoxication and metformin-induced mitochondrial dysfunction

In MB-treated cells, respiration decreased primarily when CIV was inhibited but only marginally when CIII was blocked (Fig. 2a). In cells treated with NV118, mitochondrial respiration decreased when CIII was inhibited but no further decline was seen when CIV was blocked (Fig. 2a). This was the case for cells with rotenone intoxication and also for cells with metformin-induced mitochondrial dysfunction.

## Discussion

In the present study, we demonstrated that in human platelets, the cell-permeable succinate prodrug NV118 increased mitochondrial respiration in a model of rotenone intoxication which was linked to increased coupled respiration. NV118 further improved coupled respiration significantly in a model of specific metformin-induced mitochondrial dysfunction and counteracted the phenotype that metformin had caused on lactate metabolism, as it alleviated the metformin-induced increase in lactate production in human platelets. With NV118, mitochondrial respiration increased downstream of CI and upstream of CIII, indicating CII as entry point to the OXPHOS pathway. Although the redox agent MB enhanced mitochondrial respiration downstream of CIII, it did not improve coupled respiration in human platelets in either model of drug-induced mitochondrial toxicity, nor did it mitigate the metformin-induced increase in lactate generation. In this study, human platelets were used as a substitute for more metabolically active tissues, both in regard to metformin’s mitochondrial toxicity and the treatment effect of the bypass strategies. If the counteracting effect of NV118 proves translatable to an in vivo effect, this strategy could potentially contribute to resolving the systemic energy failure associated with toxic doses of metformin and thus, correct the lactic acidosis.

**Fig. 4:**
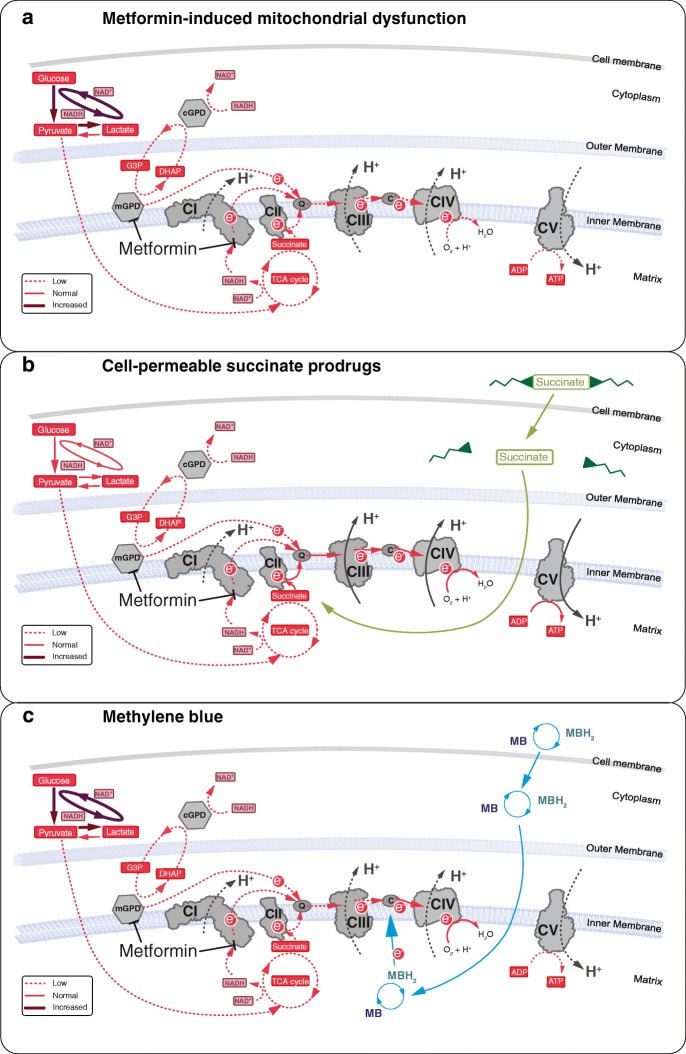
Schematic illustration of the bypass mechanism of NV118 and methylene blue in metformin-induced mitochondrial dysfunction. **a** At high concentrations, metformin induces inhibition of complex I (CI) and the mitochondrial glycerophosphate dehydrogenase (mGPD). As a result, ATP generation at complex V (CV/ATP-synthase) is decreased causing increased glycolysis to compensate for the reduced mitochondrial ATP production. Pyruvate metabolism is shifted towards lactate generation to meet the increased NAD^+^ demand, which is accompanied by intracellular and extracellular acidification. **b** Cell-permeable succinate prodrugs, such as NV118, can permeate the cell membrane independent of active transporters. Through intracellular metabolism, the succinate core is released and made available for oxidation by complex II (CII). Oxidation of succinate at CII restores downstream electron flow and mitochondrial respiration which is linked to phosphorylation (ATP generation). **c** The redox agent methylene blue (MB) is reduced by NAD(P)H-dependent dehydrogenases. The reduced form of methylene blue (MBH_2_, leucomethylene blue) then donates the electrons to cytochrome C (labeled C), recycling MB. Electron donation by MBH_2_ to cytochrome C restores downstream electron flow and mitochondrial respiration which is not coupled to phosphorylation. ADP: adenosine diphosphate, ATP: adenosine triphosphate, CIII: complex III, CIV: complex IV, cGPD: cytosolic glycerophosphate dehydrogenase, DHAP: dihydroxyacetone phosphate, G3P: glycerol-3-phosphate, NAD^+^/NADH: nicotinamide adenine dinucleotide, NADP^+^/NADPH: nicotinamide adenine dinucleotide phosphate, Q: Quinone, TCA: tricarboxylic acid

Succinate is oxidized by CII of the OXPHOS pathway (Fig. 4) [13, 15]. Succinate oxidation and subsequent electron transfer along the respiratory chain enables proton translocation across the inner mitochondrial membrane at CIII and CIV, build-up of the proton motive force, and an increase in mitochondrial ATP production. CII serves as an alternative entry point to CI for electrons to the OXPHOS pathway. Thus, the cell-permeable succinate prodrugs can bypass the CI inhibition induced by metformin (Fig. 4b). This has been demonstrated in the present study and in a study by Hinke et al. [16] in which succinate improved mitochondrial activity and rescued pancreatic β-cells from metformin-induced toxicity [16]. The improvement of OXPHOS relieved pressure from the metformin-induced increase in glycolytic ATP production with the results that less lactate was produced (Fig. 4a, b). Because NV118 lacks sufficient plasma stability, we were not able to investigate its effect in vivo. Large animal models of rotenone-induced CI-dysfunction and metformin-induced mitochondrial dysfunction like those by Karlsson et al. [17] and Protti et al. [18] would be suitable models for in vivo proof of concept of this drug class. This pharmacological bypass strategy could potentially find additional applications in intensive care. Conditions such as sepsis, traumatic brain injury, primary mitochondrial disease or drug-induced lactic acidosis are indications with a high need for further treatment development, where impaired, CI-related mitochondrial dysfunction has been described and succinate has shown success as potential treatment in vitro and in vivo [15, 19–26].

Repurposing drugs is attractive because pharmacodynamics and pharmacokinetics, safety profiles and contraindications are known. MB has been used clinically as treatment for methemoglobinemia and as an anti-malaria agent [27, 28]. Because of its potential beneficial effects on mitochondrial function, MB has recently attracted attention as treatment for neurodegenerative disorders and drug-induced side-effects [8, 11, 29, 30]. MB has been described to shuttle electrons from NAD(P)H-dependent dehydrogenase to cytochrome C (Fig. 4c) [8, 10–12, 31]. In the present study, we evaluated both bypass strategies based on immediate effects on coupled respiration and related changes in lactate metabolism. Due to the severity of MILA, there is a need for treatments acting instantly. Under these conditions, MB was unable to improve coupled respiration and alleviate the increased lactate generation associated with metformin (Fig. 4a, c). Others have previously described beneficial effects of MB on electron shuttling to the OXPHOS pathway [8, 10, 12, 32]. However, in these studies, either the oxidation of NADH at CI was evaluated in isolated, sonicated mitochondria or sub-mitochondrial particles where coupling of the electron transfer to phosphorylation pathways cannot be assessed [8, 12, 32] or it was not evaluated whether the increase in mitochondrial respiration of the fully integrated respiratory chain was linked to increased coupled respiration [10, 12]. As demonstrated in these studies, we also detected an increase in mitochondrial respiration. However, we have shown that the increased electron transport and mitochondrial respiration was not used to or is not sufficient to increase the phosphorylation pathway (Fig. 4c). By increasing CIV-linked mitochondrial respiration, MB would support proton translocation across the inner mitochondrial membrane and contribute to the proton motive force at CIV. CIV pumps less protons than complex I and III alone and thus contributes to a lesser degree to the build-up of the proton motive force which drives the phosphorylation pathway [33]. This could potentially explain the lack of beneficial effect of MB observed in the present study. On the basis of our experiments, we cannot exclude that MB potentially supports mitochondrial function through reduction of oxidative stress or stimulation of mitochondrial biogenesis and degradation of impaired mitochondria, effects that would not be immediate and might be more relevant at later stage of intervention [11, 29, 30]. MB has also been described to stabilize hemodynamic parameters through inhibition of the guanylate cyclase and has been used successfully in a small number of case reports of drug-induced shock with this objective [34].

## Conclusions

In the present study, using human platelets as model, we demonstrated that treatment with the cell-permeable succinate prodrug NV118 can ameliorate the metformin-induced impairment of the OXPHOS pathway and alleviate the associated increase in production of lactate. The redox agent MB did neither correct the inhibition of coupled mitochondrial respiration nor attenuate the increased lactate generation induced by metformin. Thus, the pharmacological bypass of metformin-induced mitochondrial dysfunction with cell-permeable succinate prodrugs presents as a promising complementary treatment strategy for patients with MILA.

## Additional files

Additional file 1: Effect of methylene blue (10 μM) and the cell-permeable succinate prodrug NV118 (250 μM) on coupled respiration after exposure to 10 mM metformin for 60 min. (PDF 42 kb)

Additional file 2: Effect of methylene blue (1 μM), the cell-permeable succinate prodrugs NV189 and NV241 (250 μM), and succinate (250 μM) on lactate production in metformin-induced mitochondrial dysfunction in human platelets. (PDF 238 kb)

## Abbreviations

C: Cytochrome C
cGPD: Cytosolic glycerophosphate dehydrogenase
CI: Complex I
CII: Complex II
CIII: Complex III
CIV: Complex IV
CV: Complex V
DHAP: Dihydroxyacetone phosphate
G3P: Glycerol-3-phosphate
MB: Methylene blue
MBH_2_: Leucomethylene blue
mGPD: Mitochondrial glycerophosphate dehydrogenase
MILA: Metformin-induced lactic acidosis
NAD^+^/NADH: Nicotinamide adenine dinucleotide
NADP^+^/NADPH: Nicotinamide adenine dinucleotide phosphate
OXPHOS: Oxidative phosphorylation
Q: Quinone
