# Supplementary material for: Bioenergetic bypass using cell-permeable succinate, but not methylene blue, attenuates metformin-induced lactate production
Source: Intensive Care Med Exp. 2018 Aug 1;6:22. doi: 10.1186/s40635-018-0186-1 (PMC6070446; doi:10.1186/s40635-018-0186-1)
Supplement: Supplementary file 1 — Effect of methylene blue (10 μM) and the cell-permeable succinate prodrug NV118 (250 μM) on coupled respiration after exposure to 10 mM metformin for 60 min. (PDF 42 kb) [file 40635_2018_186_MOESM1_ESM.pdf]

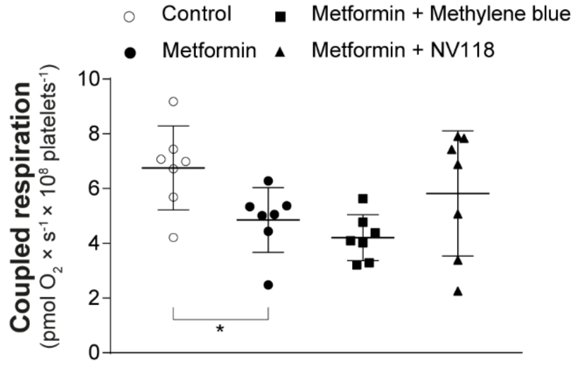

Effect of methylene blue and NV118 on coupled respiration in metformin-intoxicated human platelets. Mitochondrial respiration was measured in human platelets with mitochondrial dysfunction induced by 60 min exposure to metformin (black circle; 10 mM). After subsequent addition of methylene blue (10 µM, black square) or the cell-permeable succinate prodrug NV118 (250 µM, black triangle), mitochondrial respiration due to coupled phosphorylation, here referred to as *coupled respiration*, was evaluated by addition of the ATP-synthase inhibitor oligomycin (1 µg/ml) to block the phosphorylation pathway, and calculated as the difference in respiration before and after the inhibition of the ATP-synthase. Subsequently, the complex III inhibitor antimycin A (1 µg/ml) followed by the complex IV inhibitor sodium azide (10 mM) were added. Control experiments with both bypass strategies were performed without the addition of oligomycin to account for background drift of oxygen consumption. A vehicle control to metformin was run with each experiment (white circle). Data are expressed as individual scatter plot and mean ± SD. All experiments were performed with n=7. One-way ANOVA with Dunnet *post hoc* test was used. *$p < 0.05$, compared to metformin.
