# Supplementary material for: Bioenergetic bypass using cell-permeable succinate, but not methylene blue, attenuates metformin-induced lactate production
Source: Intensive Care Med Exp. 2018 Aug 1;6:22. doi: 10.1186/s40635-018-0186-1 (PMC6070446; doi:10.1186/s40635-018-0186-1)
Supplement: Supplementary file 2 — Effect of methylene blue (1 μM), the cell-permeable succinate prodrugs NV189 and NV241 (250 μM), and succinate (250 μM) on lactate production in metformin-induced mitochondrial dysfunction in human platelets. (PDF 238 kb) [file 40635_2018_186_MOESM2_ESM.pdf]

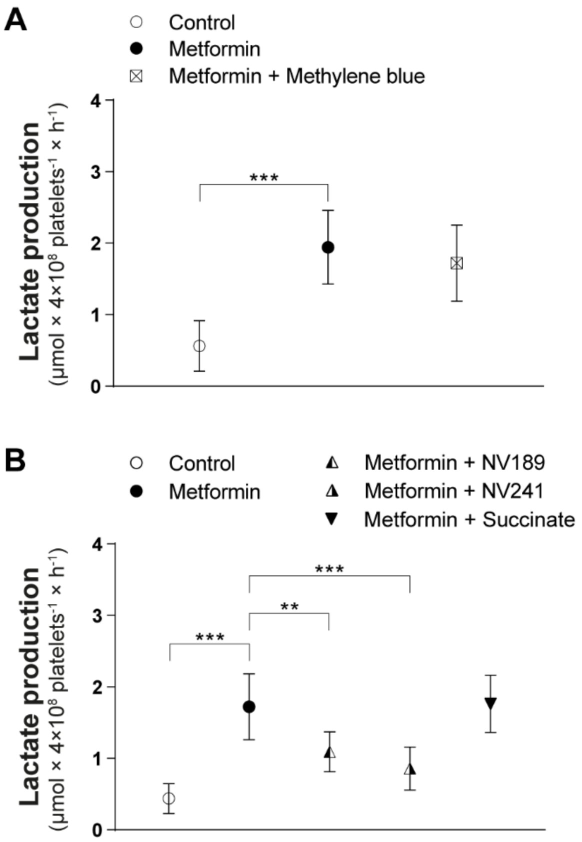

Effect of methylene blue, NV189, NV241 and succinate on lactate production in metformin-intoxicated human platelets. Lactate production of human platelets incubated with metformin (10 mM) was measured every 30 min over 4 h with or without co-treatment of **a**: methylene blue (1 µM) or **b**: NV189, NV241 or succinate (250 µM) every 30 min starting at 60 min. A vehicle control was run with each experiment (control). Data are expressed as mean ± SD. All experiments were performed with n=7, with exception of the control and metformin group of the lactate production assay run with methylene blue (n=9) (a). One-way ANOVA with Dunnet *post hoc* test was used. $^{**}p<0.01$ and $^{***}p< 0.001$, compared to metformin.
